# The stem cell session of the 7^th^ Yazd International Congress and Student Award in Reproductive Medicine held at Shahid Sadoughi University of Medical Sciences

**Published:** 2017-06

**Authors:** Zahra Borzouie, Somayyeh Sadat Tahajjodi, Behrouz Aflatoonian

**Affiliations:** 1 *Stem Cell Biology Research Center, Yazd Reproductive Sciences Institute, Shahid Sadoughi University of Medical Sciences, Yazd, Iran.*; 2 *Research and Clinical Center for Infertility, Yazd Reproductive Sciences Institute, Shahid Sadoughi University of Medical Sciences, Yazd, Iran.*; 3 *Department of Reproductive Biology, School of Medicine, Shahid Sadoughi University of Medical Sciences, Yazd, Iran.*; 4 *Department of Advanced Medical Sciences and Technologies, School of Paramedicine, Shahid Sadoughi University of Medical Sciences, Yazd, Iran. *

**Keywords:** Human embryonic stem cells (hESCs), Regenerative medicine, Reproductive medicine, Tissue engineering

## Abstract

This paper summarizes the proceedings of the stem cell session of the “7^th^ Yazd International Congress and Student Award in Reproductive Medicine” held at Shahid Sadoughi University of Medical Sciences, Yazd, Iran, on 28-30 April 2017. Here, we collected the papers of the session entitled: “Stem Cells, Good manufacturing practice, and tissue engineering”, that presented and discussed at this meeting by the international and national experts of the overlaps of the fields of stem cells and reproductive medicine, and the translation of these efforts towards practical application in regenerative medicine.

The curiosity of culturing animal cells in vitro has begun since early twenty’s (1) which can be considered as the basement of the recent novel achievements in assisted reproductive technology (ART) and stem cell biotechnology. Later then, the interest in modern biological technologies such as tissue engineering had grate increased to use in the field of cell therapy and regenerative medicine. ART in reproductive medicine, itself is a cell therapy treatment to help infertile couples to have baby. Moreover, reproductive system is one of the major sources to get pluripotent [embryonic stem cells (ESCs), embryonic germ cells (EGCs), and embryonal carcinoma cells (ECCs)], fetal [umbilical cord blood mesenchymal stem cells (UCB-MSCs), amniotic fluid stem cells], and adult [germ-line stem cells (GSCs), endometrial-derived mesenchymal stem cells (EnMSCs)] stem cells. On the other hand, stem cells biotechnology, tissue engineering, and regenerative medicine are trying to help reproductive medicine to cure cell or tissue disorders. In the stem cell session of the 7^th^ Yazd International Congress and Student Award in Reproductive Medicine, first the importance of good manufacturing practice (GMP) for translation of stem cell biotechnology to bedside and safe clinical applications was reminded and emphasized by the keynote speakers and then tissue engineering in reproductive medicine was highlighted by the experts in the field and students.

A recent 3-day, 7^th^ Yazd International Congress and Student Award in Reproductive Medicine which has coordinated with the 2^nd^ congress of reproductive genetics and 1^st^ congress of reproductive immunology was hosted by the Yazd reproductive sciences institute in Shahid Sadoughi University of Medical Sciences in the Dr. Javadi Hall of the Shahid Sadoughi Hospital, Yazd, Iran on 28-30 April 2017. Here, we collected the papers that presented and discussed at a session entitled: “Stem Cells; GMP and Tissue Engineering” that was held on 29^th^ of April. 

At the beginning of the session, Behrouz Aflatoonian (Ph.D.) presented a brief history about the progress of stem cell research in Stem Cell Biology Research Center which is based in Yazd Reproductive Sciences. According to his statement, this center has started its activities as a Stem Cell Laboratory in Yazd Research and Clinical Center for Infertility since 2006. During this time, different fields were investigated such as gonocytes (Aflatoonian *et al* presented at ISSCR 2007), TESE-derived cells (2), foreskin derived cells (Aflatoonian *et al* presented at ISSCR 2008), dental pulp stem cells (3) and application of rat bone marrow derived MSCs in treatment of stroke in animal model (4). Moreover, since 2008 (Aflatoonian *et al* presented at ISSCR 2009) an ongoing project has started to derive new human ESCs (hESCs) in the center which so far three new hESC lines (YAZD1-3) were generated (Aflatoonian *et al* unpublished data). The Stem Cell Biology Research Center has been approved by the Iranian Ministry of Health and Medical Education since 2016. To date, 9 Ph.D. projects and 14 master projects were done and in progress in the center including working with YAZD hESCs and also tissue engineering (5).

One of the challenges in IVF cycles and also for the derivation of hESCs is the number of the embryos. Marjan Omidi (M.Sc.) claimed the possibility of using the in vitro embryo twining for generation of hESC-like cells in parallel with infertility treatment. However it needs to optimize the methods (6).

Today, another most important hot topic in the field of stem cells is cell based therapies. It can be very good opportunity to treat incurable diseases. Babak Arjmand (M.D., Ph.D.) talked about clinical utilization of stem cells to translate basic sciences and protocols before starting clinical phases by bridging stem cell research into clinical trial. He stated that there are several risk factors relevant to safety issues of stem cell preparation and transplantation; include transplantation site reaction, immune responses, bio-distribution, ectopic grafting, unintended differentiation into another cell type, tumorigenicity and lack of functional characteristics. As a result, to conduct clinical stem cell transplantation trials, it is necessary to be concerned about the safety aspects of the clinical applications of the stem cells for patients (7, 8). 

In continue, Aghayan (Ph.D.) stated that in vitro manipulation of cell products requires complex laboratory procedures that increase the risk of possibly of adverse events for the recipients. He emphasized that according to the current international rules and regulations of Iranian Food and Drug Organization, cell therapy products should be manufactured under principles of GMP (9, 10). 

Prof. Harry D. Moore, discussed about their experience of derivation and maintaining clinical grade hESC lines, and focused on practical issues faced in the past and those they face in the future. Also, he talked about GMP and the process undertaken and biotechnology of hESC (11, 12).

Stem cells have long been proposed for the treatment of congenital and acquired reproductive system disorders including ovary and testis problems. Human umbilical cord blood (HUCB) are randomly harvested from fetus umbilical cord blood and are preserved for further use in liquid nitrogen. According to Seyed Nouredin Nematollahi (Ph.D.), stem cells from different adult and fetal origins including bone marrow-derived mesenchymal stem cells, Umbilical cord matrix-derived stem cells and Adipose tissue-derived stem cells were compared according to their properties. He claimed that bone marrow-derived mesenchymal stem cells and Adipose tissue-derived stem cells can be harvest from the patients and be used for the treatment of some known disease in the human and animal models. While Wharton's jelly- mesenchymal stem cells (WJ-MSCs) are immune competent and this property make them suitable for transplantation (13, 14).

One of the useful strategies to produce germ cells is to prepare adequate conditions in vitro and in vivo. Saba Behzadi (B.Sc.) reported the useful effect of bone morphogenic protein-4 and retinoic acid on differentiation of BMSCs to primordial germ like cells and spermatogonial stem like cells (15). Also, Leila Mirzaeiyan (M.Sc.) reported the successful results of production of oocyte-like cells of mouse anterior abdominal parietal peritoneum mesothelium stem cells in vitro (16).

The next part of the stem cell session was belonged to the reports about tissue engineering in reproductive system. This field is one of the hot topics in treatment of some diseases by make some new biological and non-biological scaffolds to regenerate tissues and organs. Habib Nikukar (M.D., Ph.D.) explained that regenerative medicine can offer clinicians wonderful abilities for treatment. According to his presentation there are three necessary main key elements involve in tissue engineering: cells (pluripotent and multipotent stem cells), scaffold (natural and man-made materials) and stimulant agents. He emphasized that Yazd Reproductive Science Institute is trying to have a GMP approved stem cell line production, various methods of cell therapy research and tissue engineering with special look to the regeneration of male and female reproductive systems and treatment of special challenging diseases (17, 18).

Mahdiyeh Sarabadani (M.Sc.) reported the use of mouse mesothelium layer by decellularization. This scaffold has been stated by high potentially as a three-dimensional biological scaffold, can play an effective role in improving the development of in vitro follicle culture (19). Zahra Borzouie (M.Sc.) reported the successful results of human testicular-derived cells culture from azoospermic patients on the human serum albumin -based homemade scaffolds (2). According to this study electrospun polyvinyl alcohol human serum albumin gelatin nanofibers, can be satisfactory supported scaffolds for ex vivo growth of human testicular-derived cells (20, 21).
